# Activity of CAR-T cells and bispecific antibodies in multiple myeloma with extramedullary involvement

**DOI:** 10.1038/s41408-025-01330-9

**Published:** 2025-07-30

**Authors:** Maximilian J. Steinhardt, Christoph Schaefers, Lisa B. Leypoldt, Igor-Wolfgang Blau, Marie Harzer, Xiang Zhou, Christine Riedhammer, Abdulaziz Kamili, Ricardo Kosch, Laura S. Topp, Isabel Molwitz, Nils-Ole Gross-Fengels, Yasmin Fede Melzer, Jule Artzenroth, Maximilian Al-Bazaz, Winfried Alsdorf, Max S. Topp, Johannes Duell, Julia Mersi, Johannes Waldschmidt, Carsten Bokemeyer, Hermann Einsele, K. Martin Kortüm, Katja Weisel, Leo Rasche

**Affiliations:** 1 Department of Internal Medicine II, University Hospital of Würzburg, Würzburg, Germany; 2 Department of Hematology, Oncology and Bone Marrow Transplantation with Section of Pneumology, University Medical Center Hamburg-Eppendorf, Hamburg, Germany; 3 Mildred-Scheel-Nachwuchszentrum, University Medical Center Hamburg-Eppendorf, Hamburg, Germany; 4 Bone Marrow Transplantation Department, Clinic of Haematology, Oncology and Tumorimmunology, School of Medicine, Charité University, Berlin, Germany; 5 Department of Diagnostic and Interventional Radiology and Nuclear Medicine, University Medical Center Hamburg-Eppendorf, Hamburg, Germany; 6 Mildred-Scheel-Nachwuchszentrum, University Hospital of Würzburg, Würzburg, Germany

**Keywords:** Myeloma, Chemotherapy

## Abstract

Extramedullary multiple myeloma (EMD) is associated with low response rates, short progression-free survival, and poor prognosis. CAR T cells and bispecific antibodies (bsABs) have shown efficacy in relapsed myeloma, but it remains uncertain whether one T cell redirection strategy should be preferred. We retrospectively analyzed 80 patients with EMD not adjacent to the bone treated with ide-cel, cilta-cel, teclistamab, or talquetamab at three academic centers in Germany. All patients were heavily pretreated, and a high-risk cytogenetic profile was prevalent in >41% of patients. All cohorts had a median of 5 to 7 prior lines of therapy. The vast majority of patients receiving cilta-cel, ide-cel, or teclistamab were BCMA-naive ( >88%). Response rates after CAR T cell infusion were significantly higher (100% with cilta-cel, 82% with ide-cel) than with bsABs (29% for talquetamab, 36% for teclistamab). Complete resolution of EMD was more frequent after CAR T cell therapies (50% and 41%) than after bsABs (16% and 14%). With a median follow-up of 12.2 months, median (m)PFS was not reached in patients that had received cilta-cel; mPFS was 7.3 months after ide-cel and significantly longer for both CAR T products compared to talquetamab or teclistamab (mPFS 4.0 and 2.6 months). Effective debulking therapy prolonged remissions after CAR T cell infusion compared to no debulking or no response to debulking. Visceral and soft tissue manifestations responded significantly less frequently than EMD in other locations. With significantly higher response rates, deeper remissions, and longer mPFS, our retrospective data suggest CAR T cells may provide a meaningful benefit in EMD.

## Introduction

Multiple Myeloma (MM) is a biologically heterogeneous hematologic malignancy with a variable disease course. The presence of malignant plasma cell tumors outside the bone marrow is a variant of MM defined as extramedullary disease (EMD) and is generally associated with poor outcomes. The incidence of MM with EMD is approximately 6% at initial diagnosis and has been reported to be between 6 and 20% for relapsed/refractory MM (RRMM) [1–3]. While the projected median overall survival (mOS) of patients with newly diagnosed intramedullary MM is more than 10 years [4], the presence of EMD, either at diagnosis or at relapse, is associated with poor outcome. A recent retrospective analysis from the Mayo Clinic showed an mOS of only 3.6 years when EMD was present at the time of initial diagnosis and a survival of approximately 0.7 years for patients with secondary EMD [5]. The recent real-world LocoMMotion study confirms a dismal mOS of only 8 months for patients with secondary EMD [6, 7].

T-cell redirecting bispecific antibodies (bsABs) and CAR-T cells have shown impressive response rates and progression-free survival (PFS) compared to the previous standard of care in RRMM patients [8–11], and there was hope that the same might hold true for EMD [12, 13]. Indeed, the pivotal KarMMa trial reported an overall response rate (ORR) of over 70% and a survival benefit for EMD patients treated with idecabtagene vicleucel (ide-cel), but this analysis subsumed not only EMD but also paraskeletal disease, making interpretation difficult [14]. In a retrospective analysis, 11 US centers reported outcomes of 84 EMD patients treated with ciltacabtagene autoleucel (cilta-cel), another BCMA-directed CAR-T cell therapy [15], and found an ORR of 52%, an mPFS of 5.3 months, and an mOS of 14.8 months, with overall inferior outcomes compared to patients without EMD [16]. Similarly, in two other recent real-world analyses of 134 and 255 patients receiving cilta-cel, EMD was associated with significantly lower ORR and both shorter PFS and OS compared to non-EMD patients [17]. Nevertheless, the results were promising compared to historical EMD cohorts.

For GPRC5D-targeted bsAB talquetamab, the MonumenTAL-1 study reported an ORR of over 40% for true EMD (i.e., not adjacent to the bone) [18]. With the BCMA-targeted bsAB teclistamab, the ORR was 36% in the pivotal MajesTEC-1 study [19] and 47% in a real-world analysis [20]. However, multivariate analysis identified EMD as an independent risk factor for inferior ORR and PFS. This was recently confirmed in a large real-world cohort with an mPFS of only 2.1 months for EMD patients [21].

With multiple CAR T cell products and bsABs targeting different antigens available, it remains an open question which should be preferred in the treatment of EMD. To address this question, we analyzed the outcomes of 80 EMD patients with RRMM treated with novel immunotherapies in three academic centers in Germany.

## Methods

### Ethics approval and consent to participate

This study was approved by the ethics committee (Medizinische Ethikkommission an der Julius-Maximilians-Universität Würzburg, 20240301-01) in accordance with the Declaration of Helsinki. Informed consent was obtained from all participants.

### Study cohort

We retrospectively analyzed data from patients with EMD not adjacent to the bone who were started on therapy with bsABs or CAR T cell therapies from three academic centers in Germany from 09/21 to 09/24. Patients were included only if both serologic assessment and EMD imaging results were available less than 90 days prior to initiation of lymphodepleting therapy or bsAB initiation and at follow-up after at least three months or at the time of progression. Patients who received bridging with bsABs to CAR-T were excluded. We analyzed demographics, laboratory parameters, bone marrow plasma cell (BMPC) infiltration, PFS and OS outcomes, cytogenetics, and prior therapies.

### Data assessment

Treatment response was assessed using the International Myeloma Working Group (IMWG) criteria [22]. Patients with oligo- or asecretory disease were not analyzed for serologic response.

MM was considered EMD only if visceral or soft tissue lesions were not adjacent to bone. EMD response was measured by either MRI, CT, or FDG-PET/CT according to each center’s policy. EMD response was categorized as complete response (CR), partial response (PR), stable disease (SD), or progressive disease (PD) according to IMWG criteria [22]. Mixed responses were classified as SD. Moreover, the tissue-specific response rate of EMD was recorded. High-risk cytogenetics were defined according to the R-ISS criteria [23].

### Statistical analysis

Groups were compared using the chi-squared test. PFS and OS analyses were performed from CAR-T cell infusion or first bsAB treatment using Kaplan–Meier analysis and compared using the log-rank test.

Continuous data are described as median and range. Univariable and multivariable Cox and logistic models were fitted to evaluate the influence of possible prognostic factors on EMD remission, PFS, and OS. To illustrate the results of the Cox models, hazard ratios were calculated. All statistical analyses were performed using GraphPad Prism 9®, JMP Edition 18, and SPSS Statistics® version 29. All statistical analyses were 2-sided. *p* Values ≤ 0.05 were considered statistically significant.

## Results

### Baseline characteristics

We included 80 RRMM patients with EMD with a median age of 53 years treated with either cilta-cel (*n* = 14), ide-cel (*n* = 22), talquetamab (*n* = 28), or teclistamab (*n* = 16). EMD locations were most common within muscle tissue (40%), skin (34%), pleura (31%), and retroperitoneum (24%). The median number of reported extramedullary lesions was two (range 1–16) in all groups. We excluded *n* = 3 patients with concurrent plasma cell leukemia from this analysis.

Overall, disease and patient characteristics were similar between the four groups. Baseline ferritin and bone marrow plasma cell infiltration were not statistically different. Patients had a median of 5–7 prior lines of therapy and were exclusively triple-refractory. More than 67% were penta-refractory with equal distribution over all groups (*p* = 0.92), reflecting the authorization status of the therapeutic agents at the time. The population was enriched for high-risk cytogenetics (54% of patients with FISH analyses available for 74 out of 80 patients). Due to CAR T manufacturing times, most CAR T cell patients received debulking therapy (85% before cilta-cel, 90% before ide-cel), with PACE combinations (cisplatin, doxorubicin, cyclophosphamide, and etoposide, *n* = 4) and pomalidomide, bortezomib, doxorubicin, dexamethasone, and daratumumab (*n* = 8) as most frequent debulking regimens. As a result of the higher rate of debulking therapy, the rate of disease control at therapy initiation was significantly higher in the CAR T cell cohorts, with 41% and 36% of all patients with progressive disease at the time of cilta-cel or ide-cel infusion, respectively. Radiation was performed in three patients. In these cases, target lesions were not included in the response analysis. Intrathecal therapy was given in one case before treatment with ide-cel, but without resolution of plasma cell infiltration of the cerebrospinal fluid. There was no intrathecal therapy given during systemic therapy. Most patients receiving cilta-cel, ide-cel, or teclistamab were BCMA-treatment naïve (>88% across all groups, *p* = 0.69). A comparison between the cohorts is given in Table 1.

**Table 1:** Baseline characteristics at therapy initiation.

Baseline	Cilta-cel (*n* = 14)	Ide-cel (*n* = 22)	Talquetamab (*n* = 28)	Teclistamab (*n* = 16)	*p*
Male/female (%)	77/23	68/32	61/39	65/35	0.76
Age (years; median, range)	67 (41–74)	58 (46–81)	55 (35–80)	60 (36–82)	0.35
Prior LOT (median, range)	5 (3–7)	6 (3–8)	6 (4–19)	7 (3–17)	**0.05**
Triple/penta-refractory (%)	100/77	100/64	100/75	100/77	0.92
High risk (%)	62	45	52	67	0.58
Mean Ferritin (µg/l)	1976	830	1400	2013	0.69
Median bone marrow plasma cells (%, range)	0 (0–80)	5 (0–80)	0 (0–90)	0 (0–90)	0.57
Median time between Dx and EMD (years)	2.6	4.4	3.9	3.0	0.47
Median time between MM Dx and Tx (years)	4.5	6.8	6.5	4.5	0.64
Median time between EMD Dx and Tx (years)	1.3	0.7	1.3	1.5	0.88
Prior BCMA-targeted therapy (%)	8	15	25	18	0.69^a^
Progression to debulking/prior Tx (%)	36	45	79	100	**<0.01**

*BMPC* bone marrow plasma cells, *LOT* line of therapy, *EMD* extramedullary disease, *Dx* diagnosis, *CR* complete remission, *Tx* therapy, *PFS* progression-free survival. Significance was tested using the log-rank test. Significant results are printed in bold.

^a^Excluding talquetamab cohort as non BCMA targeting Tx.

### Response

**Fig. 1:**
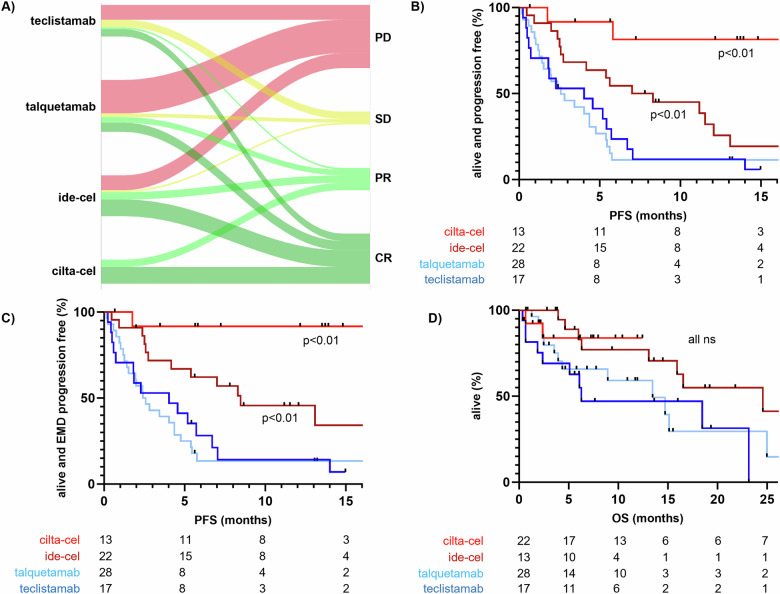
Response, progression-free, and overall survival of EMD patients treated with ide-cel, cilta-cel, talquetamab, or teclistamab. **A** Visual representation of response patterns in regard to therapy. **B** PFS (serologic and EMD combined). **C** PFS (EMD only). **D** OS analysis by therapy.

EMD response rates in patients receiving CAR T cells were significantly higher (100% for cilta-cel, 82% for ide-cel) than with bsABs (29% for talquetamab, 36% for teclistamab, *p* < 0.0001, see Fig. 1A). Complete resolution of EMD was also significantly more frequent with CAR T-cell therapies (69% and 41%, respectively) than with bsABs (18% and 24%, respectively, *p* = 0.001). With a median follow-up of 12.2 months, median PFS was not reached in patients after cilta-cel and was significantly longer with ide-cel at 7.6 months when compared to talquetamab and teclistamab (2.6 and 4.0 months, respectively, *p* = 0.03), (Fig. 1B). The time to EMD recurrence was longer only after CAR T-cell therapy (not reached for cilta-cel, 8.5 months after ide-cel) or did not occur at all during follow-up (Fig. 1C). Median OS was not reached for patients receiving cilta-cel, and amounted to 24.6 months for ide-cel, 13.5 months for talquetamab, and 6.3 months for teclistamab (ns, Fig. 1D). Details on survival analyses are given in Table 2.

**Table 2:** Response to therapy.

Survival analysis	Cilta-cel (*n* = 14)	Ide-cel (*n* = 22)	Talquetamab (*n* = 28)	Teclistamab (*n* = 16)
EMD response (≥PR, %)	100	82	29	36
EMD: CR (%)	69	41	18	24
EMD relapse at progression (%)	50	61	96	93
Median PFS (months)	NR	7.6	2.6	4.0
Median OS (months)	NR	24.6	13.5	6.3
Median follow-up (months)	12.2	16.5	8.7	6.1

EMD extramedullary disease, *PFS* progression-free survival, *PR* partial response, *Dx* diagnosis, *CR* complete response, *OS* overall survival. The estimated observation based on the median time to censoring (reverse KM) showed no differences with a median follow-up of 10.02 ± 1.16 months.

**Fig. 2:**
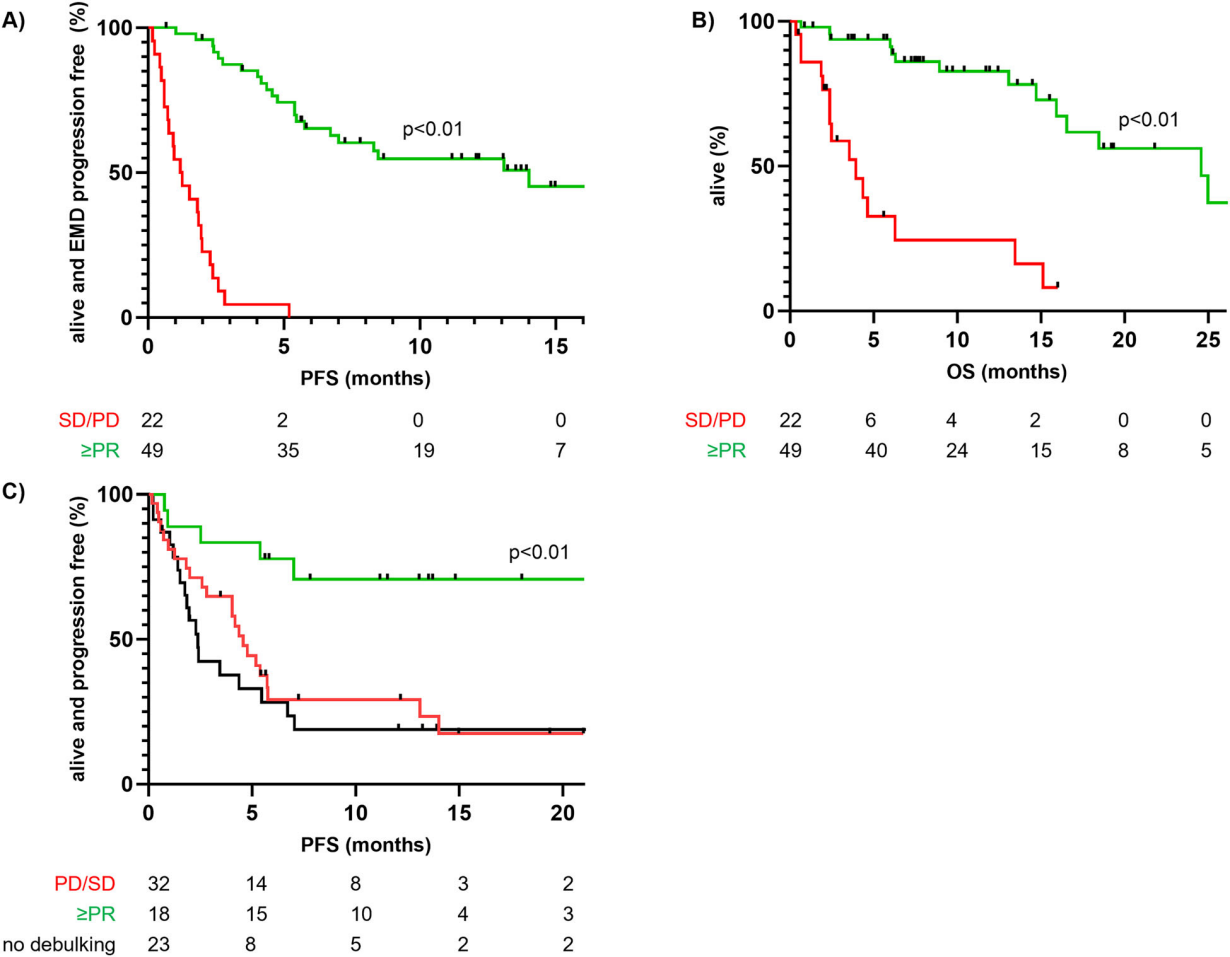
Predictors of progression-free and overall survival after T-cell redirecting therapy. **A** EMD PFS analysis by serologic response. **B** OS by serologic response. **C** EMD PFS by response to bridging.

A mixed response pattern between serologic markers and EMD was common. 17% of all serologic responders showed stable or progressive EMD, but there were no patients with an EMD response who did not also show serologic improvement. Thus, serologic response was strongly associated with EMD remission (*p* < 0.01). There were 10 cases of a secretory progression requiring confirmation via whole body MRI or PET/CT scans, bone marrow biopsies revealed that 75% of patients had macrofocal relapse. Successful debulking therapy (PR or better) was associated with longer PFS in patients with EMD treated with CAR T cells. In fact, these patients had the longest mPFS (not reached at a median follow-up of 11.2 months, *p* < 0.01, see Fig. 2A). Patients who did not respond to CAR-T cell therapy had a dismal prognosis with a median OS of 3.5 months (see Fig. 2B). Failure of debulking therapy resulted in a similar mPFS to those who did not receive therapy before T-cell redirecting therapy (2.4 vs. 4.6 months, ns, see Fig. 2C).

### Prognostic factors

**Fig. 3:**
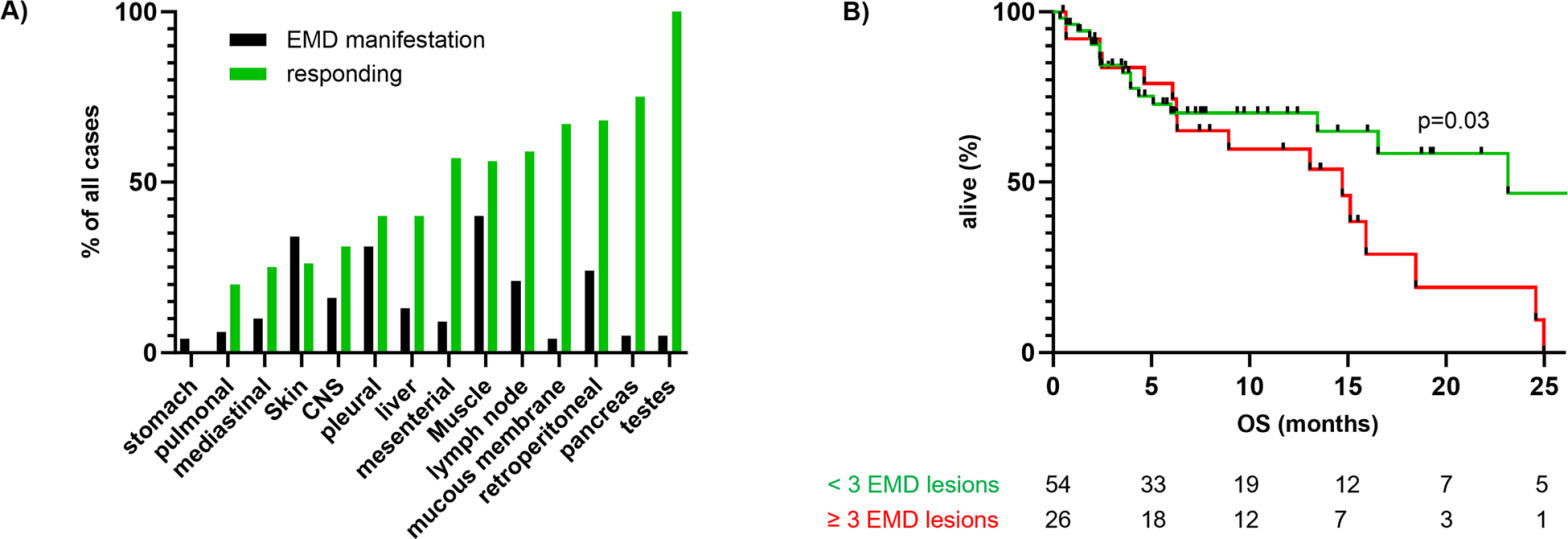
EMD response by localization, and number of EMD lesions. **A** Analysis of EMD response by localization. **B** Analysis of EMD response by number of lesions.

We further addressed the question of whether there is a subgroup of EMD patients who exhibit particularly adverse outcomes even when treated with novel immunotherapies. Here, regardless of treatment modality, parenchymal manifestations were less likely to respond than skin, soft tissue, and lymph node lesions (ORR 39% vs. 48%, see Fig. 3A) and relapsed more often within our FU period (81.3 vs. 57.6%). Patients with a higher number (≥3) of extramedullary lesions had worse outcomes regardless of localization and treatment choice (*p* = 0.04, see Fig. 3B).

Univariate risk analyses showed a correlation between PFS and the number of prior LOT (*p* = 0.05), especially prior BCMA-targeted therapy (*p* = 0.01). This was confirmed by multivariate analyses. In this context, it is important to note that patients in the bsABs cohorts had received a slightly higher number of prior LOT compared to the other groups (median 6–7 vs. 5–6 in CAR T patients, *p* = 0.05). Moreover, progression to last therapy was strongly associated with shortened PFS (*p* < 0.01) and EMD PFS (*p* = 0.01), but multivariate analysis showed no correlation to OS. There was a trend for inferior PFS and OS in patients with higher BMPC and ferritin at therapy initiation as reported previously [24], but significance was not met. Relative risks are given in Table 3.

**Table 3:** Proportional hazards fit.

	Overall PFS		EMD PFS		OS	
	HR	*p*	HR	*p*	HR	*p*
Male gender	1.14	0.87	0.78	0.80	0.07	0.19
Age	1.03	0.29	1.02	0.48	1.19	**<0.01**
Number of prior LOT	1.62	**0.05**	1.74	**0.03**	3.69	**<0.01**
Refractory to last LOT	9.06	**<0.01**	26.70	**0.01**	2500	**0.05**
Penta-refractory	4.48	0.23	5.44	0.17	42.90	**0.04**
High-risk FISH	1.36	0.69	1.24	0.80	3.86	0.16
Ferritin	1.00	0.31	1.00	0.34	1.0	0.09
BMPC	1.02	0.10	1.01	0.19	1.0	0.51
Time Dx to EMD	0.25	0.24	0.71	0.80	0.83	0.61
Time MM Dx to Tx	3.94	0.25	1.41	0.81	1.86	0.60
Time EMD Dx to Tx	0.25	0.24	0.70	0.80	0.84	0.61
Prior BCMA-targeted therapy	7.23	**0.01**	5.37	0.07	21.46	**0.03**
Number of EMD lesions	1.26	0.27	1.08	0.76	2.1	**0.04**

*LOT* line of therapy, *BMPC* bone marrow plasma cells, *EMD* extramedullary disease, *PR* partial remission, *Dx* diagnosis, *CR* complete remission, *Tx* therapy, *PFS* progression-free survival. There was no significant correlation between any of the y-listed categories and response rates. Significant results are printed in bold.

Moreover, we found a striking difference in the relapse patterns between the cohorts: 35% of patients after CAR T cell therapy showed serological progression only, while maintaining sustained remission of EMD. In contrast, following treatment with bsABs, only 5% of relapses occurred without EMD recurrence. Notably, 4 out of 7 CNS lesions resolved after CAR T-cell therapy without the need for additional intrathecal chemotherapy or radiotherapy, whereas no therapeutic responses were observed in the 7 CNS cases treated with bsABs.

## Discussion

Our analysis shows that all novel T-cell recruiting immunotherapies have significant activity in RRMM patients with EMD. Treatment with multiple different immunotherapies may be required to achieve the greatest survival benefit in this vulnerable population. In direct comparison, our study suggests that CAR T-cell therapy leads to favorable outcomes with regard to depth of response and survival compared to bsABs.

This notion is supported by a recent report on the efficacy of ide-cel in RRMM patients, including those with true EMD (*n* = 84), which was the first to report a meaningful mPFS of 5.3 months, providing hope that CAR-T-cell therapies could substantially improve outcomes of EMD [16].

Significant caveats must be considered when comparing the two modalities to avoid confounding, as CAR-T cell procedures require some degree of disease control prior to application. This may select patients with less aggressive disease, response to debulking therapy, or earlier relapses, as reflected in the higher number of prior LOT and higher rate of progression prior to therapy in our bsAB cohorts. Consequently, the more heavily pretreated status of patients receiving bsABs may have contributed to a potentially lower therapeutic activity in the EMD setting. However, penta-refractory status, number of EMD lesions, baseline ferritin, bone marrow plasma cells infiltration, and time from EMD and MM diagnosis at baseline were similar between cohorts. This suggests no major difference in the timing of treatment initiation and disease aggressiveness. For CAR T cell therapies specifically, the debulking effect of effective bridging therapies appears to be key to prolonging remissions.

There are several hypotheses as to why CAR-T cells appear to be more effective in the EMD setting than bsABs. The expansion of CAR T cells after infusion could result in an advantageous effector-target ratio when compared to bsABs, especially since some EMD tumors are depleted of autologous T cells [25]. In contrast, bsABs activate the patient’s own T cells, which are either depleted or show an exhausted phenotype, as recently shown by our group and others [26, 27]. This may in part explain the discordant response kinetics in our study, showing higher serologic response rates than EMD responses, supporting a setting in which a diffuse concomitant bone marrow infiltration can be cleared by bsABs while the solid EMD manifestations persist due to T-cell scarcity and exhaustion [28]. Serum levels of soluble (s)BCMA play a role in this complex response biology because BCMA-directed bsABs bind to sBCMA and may not reach their target cells. In contrast, CAR-T cells have a comparatively lower affinity to sBCMA and are, therefore, less susceptible to high sBCMA serum levels [25]. These mechanisms combined may explain the difference in response rates, depth of remission, EMD resolution, and PFS. However, this does not explain the decreased activity of talquetamab in EMD. Though first results from the phase 1b RedirecTT-1 trial combining talquetamab and teclistamab and thereby targeting both GPRC5D and BCMA show unprecedented activity in triple-exposed patients with EMD with an ORR of 85.6% and 28.6% ≥CR-rate [29] and seem promising in overcoming some of the limitations of bsAB monotherapy.

Interestingly, after CAR T-cell therapy, 35% of patients in our study relapsed only serologically, but had sustained EMD remission. In contrast, after bsAB therapy, only 5% of all cases showed this relapse pattern. These results suggest that T-cell activity in EMD lesions is significantly higher with CAR T cells compared to bsAB redirection. Thus, CAR T cell therapy can eradicate solid lesions, but it still has difficulty completely clearing the bone marrow. This is in line with observations recently made in mouse models of breast cancer treated with novel immunotherapies [28].

We show that both multi-site disease and visceral involvement correlate with inferior outcomes, independent of immunotherapeutic modality. Other studies hypothesized that high tumor burden, which is associated with an increased number of extramedullary lesions, may inhibit T-cell efficacy due to accelerated exhaustion [30, 31]. In our study, we accordingly found that patients who received effective debulking therapy followed by CAR T-cells had a longer PFS compared to patients without application or response to debulking therapy. This suggests the PFS benefit is not a function of poor prognosis due to a more aggressive phenotype, but that an effective debulking therapy is key to prolonging remissions after CAR T infusion. Which debulking therapy is most effective remains to be elucidated, but the importance of reaching short-term responses could argue for intensive therapies.

A dedicated analysis of imaging patterns and time points after immunotherapy is an important next step, as some of the lesions may not represent residual disease but pseudoprogression during focal expansion of T cells [32, 33]. Moreover, the rate of macrofocal relapse was high, and all cases of asecretory progression were only seen in whole-body MRI or PET/CT scans. Therefore, our data support the recently proposed EMD standardization of functional imaging [34].

Limitations of our data lie in the nature of retrospective analyses. We have adjusted for some potential confounders, but in the absence of randomization, these are inherent in the choice of therapy. OS events were rare due to limited follow-up. In addition, results may be underpowered due to small sample sizes in subgroup analyses.

## Conclusion

Both CAR T cells and bsABs show efficacy in EMD. Acknowledging the limitations of retrospective analyses, our data suggests that in this setting, CAR T cells may be superior. They are associated with a higher rate of EMD resolution and longer mPFS in the case of relapse. In EMD, the debulking effect of effective bridging therapies is key to prolonging remissions after CAR T infusion. However, the negative prognostic impact of EMD cannot be fully overcome with CAR T-cell therapies, highlighting the ongoing knowledge and treatment gap for this challenging and increasingly common manifestation of MM.

## Data Availability

All data will be provided by the authors upon reasonable request.
