# Correction: Activity of CAR-T cells and bispecific antibodies in multiple myeloma with extramedullary involvement

**DOI:** 10.1038/s41408-025-01403-9

**Published:** 2025-10-31

**Authors:** Maximilian J. Steinhardt, Christoph Schaefers, Lisa B. Leypoldt, Igor-Wolfgang Blau, Marie Harzer, Xiang Zhou, Christine Riedhammer, Abdulaziz Kamili, Ricardo Kosch, Laura S. Topp, Isabel Molwitz, Nils-Ole Gross-Fengels, Yasmin Fede Melzer, Jule Artzenroth, Maximilian Al-Bazaz, Winfried Alsdorf, Max S. Topp, Johannes Duell, Julia Mersi, Johannes Waldschmidt, Carsten Bokemeyer, Hermann Einsele, K. Martin Kortüm, Katja Weisel, Leo Rasche

**Affiliations:** 1 Department of Internal Medicine II, University Hospital of Würzburg, Würzburg, Germany; 2 Department of Hematology, Oncology and Bone Marrow Transplantation with Section of Pneumology, University Medical Center Hamburg-Eppendorf, Hamburg, Germany; 3 Mildred-Scheel-Nachwuchszentrum, University Medical Center Hamburg-Eppendorf, Hamburg, Germany; 4 Bone Marrow Transplantation Department, Clinic of Haematology, Oncology and Tumorimmunology, School of Medicine, Charité University, Berlin, Germany; 5 Department of Diagnostic and Interventional Radiology and Nuclear Medicine, University Medical Center Hamburg-Eppendorf, Hamburg, Germany; 6 Mildred-Scheel-Nachwuchszentrum, University Hospital of Würzburg, Würzburg, Germany

**Keywords:** Myeloma, Chemotherapy

Correction to: *Blood Cancer Journal* 10.1038/s41408-025-01330-9, published online 30 July 2025

Following the publication of this article, an error was noted in the text of the introduction.

The following paragraph:

“T-cell redirecting bispecific antibodies (bsABs) and CAR-T cells have shown impressive response rates and progression-free survival (PFS) compared to the previous standard of care in RRMM patients [8,9,10,11], and there was hope that the same might hold true for EMD [12, 13]. Indeed, the pivotal KarMMa trial reported an overall response rate (ORR) of over 70% and a survival benefit for EMD patients treated with idecabtagene vicleucel (ide-cel), but this analysis subsumed not only EMD but also paraskeletal disease, making interpretation difficult [14]. In a retrospective analysis, 11 US centers reported outcomes of 84 EMD patients treated with ciltacabtagene autoleucel (cilta-cel), another BCMA-directed CAR-T cell therapy, and found an ORR of 52%, an mPFS of 5.3 months, and an mOS of 14.8 months, with overall inferior outcomes compared to patients without EMD [16]. Similarly, in two other recent real-world analyses of 134 and 255 patients receiving cilta-cel, EMD was associated with significantly lower ORR and both shorter PFS and OS compared to non-EMD patients [17]. Nevertheless, the results were promising compared to historical EMD cohorts.”

Is replaced with:

“T-cell redirecting bispecific antibodies (bsABs) and CAR-T cells have shown impressive response rates and progression-free survival (PFS) compared to the previous standard of care in RRMM patients [8,9,10,11], and there was hope that the same might hold true for EMD [12, 13]. Indeed, the pivotal KarMMa trial reported an overall response rate (ORR) of over 70% and a survival benefit for EMD patients treated with idecabtagene vicleucel (ide-cel), but this analysis subsumed not only EMD but also paraskeletal disease, making interpretation difficult [14]. In a retrospective analysis, 11 US centers reported outcomes of 84 EMD patients, and found an ORR of 52%, an mPFS of 5.3 months, and an mOS of 14.8 months, with overall inferior outcomes compared to patients without EMD [16]. A recent real-world analysis of in 236 patients treated with ciltacabtagene autoleucel (cilta-cel), another BCMA-directed CAR-T cell therapy, reported that true EMD was associated with a significantly lower ORR, as well as shorter PFS and OS, compared to non-EMD patients[17]. Nevertheless, the results were promising compared to historical EMD cohorts.”

Citation 15 and 18 have also been removed.

The original article has been corrected.

